# The effect of reading literary fiction on the theory of mind skills among persons with schizophrenia and normal controls

**DOI:** 10.3389/fpsyt.2023.1197677

**Published:** 2023-06-07

**Authors:** Judit Fekete, Zsuzsanna Pótó, Eszter Varga, Dóra Hebling, Márton Herold, Noémi Albert, Borbála Pethő, Tamás Tényi, Róbert Herold

**Affiliations:** ^1^Department of Languages for Biomedical Purposes and Communication, Medical School, University of Pécs, Pécs, Hungary; ^2^Institute of Physiotherapy and Sport Science, Faculty of Health Sciences, University of Pécs, Pécs, Hungary; ^3^Department of Pediatrics, Medical School, University of Pécs, Pécs, Hungary; ^4^Department of Psychiatry and Psychotherapy, Medical School, University of Pécs, Pécs, Hungary

**Keywords:** theory of mind (ToM), mental state reasoning, literature, reading, Short Story Task, schizophrenia

## Abstract

**Introduction:**

Recent research data suggest that theory of mind (ToM) skills may improve after reading literary fiction. However, beside this short term favorable effect, regular long-term reading of literary fiction may also support ToM development or may improve ToM performance. The presence of impaired ToM abilities is well-documented in schizophrenia; however, the role of reading in these deficits is unknown. In the present study our aim was to assess the effect of prior reading experiences on theory of mind performance in patients with schizophrenia, and in healthy controls.

**Materials and methods:**

ToM assessment was done with the Short Story Task, which is based on the interpretation of a Hemingway short story. After reading the short story, questions were asked in an interview format regarding comprehension, explicit and implicit ToM skills, then comparative analysis of schizophrenia patients was performed (*n* = 47) and matched to a normal control (*n* = 48) group concerning deficits of ToM abilities. Participants were also stratified according to their prior reading experiences.

**Results:**

Previous reading experience was associated with better comprehension and explicit ToM performance both in patients with schizophrenia, and in healthy controls. However, the explicit ToM performance of patients with prior reading was still weaker compared to healthy controls with reading experiences. Path model analysis revealed that reading had a direct positive effect on ToM, and an indirect effect through improving comprehension.

**Conclusions:**

Prior reading experience is associated with better ToM performance not just in healthy controls but also in patients living with schizophrenia. Previous reading experience also improves comprehension, which in turn has a favorable impact on ToM. Our results support the idea that literary fiction reading may have a therapeutic potential in the rehabilitation of schizophrenia.

## Introduction

As part of the adaptive response to ever-expanding social relationships, the ability to relate visible behavior to mental states, and thus to see ourselves from the outside and others from the inside, has evolved, and is called theory of mind (ToM; also known as mentalizing), the ability to represent the mental states of others (1). It helps us to recognize the meaning behind other people's sentences, explain actions, to understand irony and to predict their behavior. There are two core levels of ToM, so we can talk about a fast, automatic, non-verbal, unconscious implicit ToM and a slower, reflective, verbal, and conscious explicit ToM (2). Studies using ToM measurements indicate that deficits in ToM are associated with several psychopathological states including autism and schizophrenia (3, 4), just as better performance on tests of ToM relates to more prosocial (5) and effective social interactions (6, 7).

The effect of fiction has been a topic of interest since the time of Aristotle, but it is only in recent decades that it has become a focus of psychological research. What does reading do to us, what is its mechanism of action, what makes it enjoyable and useful? A plausible explanation is that this is due to its effect on ToM (8–10). Research has revealed that familiarity with literary fiction authors, a good proxy measure of actual reading habits, correlates with scores on a test of ToM (10). Numerous studies have been carried out on the relationship between ToM and text comprehension. Kim (11, 12), Boerma et al. (13), and Atkinson et al. (14) have all studied the relationship between ToM and reading/text comprehension among school children. In a significant proportion of these papers ToM was considered one of several predictor variables for text comprehension, clearly providing a valuable approach to understanding ToM more closely. In addition a large volume of research evidence shows a positive relationship between reading literature and ToM or empathy (8, 10, 15).

While there is ample evidence that reading literary fiction is associated with increased emotional understanding of others, until the findings of Kidd and Castano (9, 16) and Black and Barnes's (17) fit was not clear whether this effect was unique to written fiction, or whether the complexity and intricacy of the characters and relationships in the fictional story mattered more than the medium itself. When readers use their ToM abilities to imagine what the characters might be feeling or thinking, this improves their mental model of the story and contributes to their reading comprehension (14, 18–20). In 2013 Kidd and Castano tested their hypothesis that reading literary fiction would lead to improved ToM performance in five experiments, four of which directly contrasted literary and popular fiction or non-fiction. Consistent with the hypothesis, results revealed higher ToM performance among participants assigned to read literary fiction (16, 17, 21, 22). Zushine (23) argues that this is due to the property of literary fiction to draw rounded, 3-dimensional, complex characters, encouraging the reader to constantly shape, adjust and consider multiple interpretations of the characters' mental states, in contrast to the stereotypical, more schematic characters of genre fiction. The fundamental difference between fiction and genre literature is that while the former focuses on the complexity and diversity of the characters, the latter concentrates more on familiar themes, patterns and plot. In contrast, Tamir and colleagues in 2016 argued that biographies, memoirs and narrative journalism are forms of non-fiction literature that can be read to similar effect (24). Current research shows that playing non-violent video games, that focus on storytelling and role-playing (25), or watching quality TV dramas also improves ToM (17). The emergence and development of ToM can best be fostered in a social environment where there is more opportunity for interactions that are not based on rigid and schematic social roles.

Reading has also been linked to a short-term effect on ToM and a long-term effect on understanding mental states. A subsequent series of large-scale correlational studies further demonstrated the reliable finding that lifetime exposure to fiction positively predicts ToM performance (10, 15, 26), and that exposure to fiction is a determinant of ToM performance (16).

More recently literary fiction has also been used to assess ToM abilities, based on the studies mentioned above. A new innovative approach by Dodell-Feder's (27) tested these skills by interpreting short stories using a structured interview. Short Story Task (SST) proved to be a suitable tool for measuring ToM without ceiling effect and to show concurrent validity with other ToM tests in healthy subjects.

ToM impairment is a core feature of schizophrenia. Several publications, including meta-analyses and systematic reviews have confirmed the widespread ToM impairments in schizophrenia (4, 28–31). ToM deficits are observed in both the acute phase of schizophrenia, and also in remission (4). Patients with schizophrenia show abnormal brain activation during ToM tasks (32). ToM impairments are not only present in the first episode schizophrenia, but also in high risk individuals before the onset of the disease (30). Impaired performance has also been shown in unaffected first degree relatives (30, 33). Patients with schizophrenia exhibit deficits in both the cognitive and the affective components of ToM (34). They have consistently exhibited low performance in several different types of assessment tools (4). Although the vast majority of studies have examined explicit ToM skills, a recent meta-analysis also found deficient implicit ToM performance in schizophrenia albeit to a lesser extent (35).

Despite ToM impairments in schizophrenia, there is a significant habitual limitation of ToM assessment: results often show a ceiling effect, making it difficult to detect subtle individual differences (36). However, a more detailed picture of the ToM impairements would be essential for planning a personalized approach to improve social cognition in schizophrenia. Accordingly, as an extension to Dodell-Feder's study we (36) examined their method comparing healthy participants with persons living with schizophrenia finding this method suitable for measuring ToM. Our results lined up with the original Dodell-Feder et al. findings that reading fiction could be used as an assessment tool for ToM skills in persons with schizophrenia. Patients performed more poorly in SST compared to healthy controls. The SST lacked the ceiling effect, and it was sensitive to explore the individual differences in ToM performance, and so it can be useful in planning psychosocial interventions.

Since the publication of SST, it has also been applied to assess individuals at high clinical risk for psychosis (37), and most recently, SST was successfully used in subjects with autism spectrum condition (38).

However, based on the aforementioned research data, we can also hypothesize that reading literary fiction may enhance ToM skills (15, 17, 22, 39), which in turn suggests that it may be a potential means of improving ToM deficits in certain psychopathological states, such as schizophrenia (21, 36) through remediation. Based on these findings, we reanalysed our SST data (36) focusing on the effect of previous literary fiction reading experience on SST performance.

To do so, we focused on the first open-ended question of the SST, which queries about previous experiences of reading literary fiction and whether participants referred back to or mentioned previous reading experiences during the survey.

In this current study we hypothesized that participants in both experimental groups (schizophrenia and control groups) with previous fiction reading experiences would outperform participants in the text comprehension questions and would have higher ToM scores. We also hypothesized that the performance of the previous readers in the clinical group would be still significantly impaired compared to that of the non-clinical group. We also presumed that ToM skills may be more preserved in subjects with schizophrenia when there's prior literary reading experience compared to non-reading patients so this could play an important role in the rehabilitation process for patients living with schizophrenia.

## Method

### Participants

Two groups of participants, a clinical (patients with schizophrenia in remission) and a non-clinical group (control group, CG) were recruited for the study. The group of 47 patients (schizophrenia group, SG) was recruited from the outpatient clinics from various cities in Southern Hungary by their respective psychiatrists. Persons with schizophrenia in remission (*n* = 47) and healthy controls (*n* = 48) were assessed and compared. Both groups consisted of native Hungarian-speaking individuals, and the age of all subjects were older than 18 (Mean age in CG: 43.88, SD = 19.38); mean age in SG: 43.64, SD: 11.30). The clinical participants had to have been diagnosed with schizophrenia confirmed by a psychiatrist, according to the DSM-5 diagnostic criteria. Diagnosis was also confirmed by Module B and C of SCID-5 (40). Patients were on maintenance antipsychotic treatment (first and second generation antipsychotics or a combination of two) and were all clinically stable. In addition to antipsychotics, some patients were also treated with adjunctive pharmacotherapy (benzodiazepines, anticholinergics, or antidepressants). Patients had no changes in their medication in the previous 6 months prior to the study. They had to obtain a score of ≤ 3 on the scale of positive and negative symptoms (PANSS) and remission was confirmed with the eight items (P1, P2, P3, N1, N4, N6, G5, and G9). The 48 non-clinical control subjects were recruited from the community through word of mouth recruitment and in discussions with groups of individuals. They had to have a sociodemographic profile (age, education, and family education) comparable to that of the clinical group. Inclusion criteria for the controls were the following: no evidence of substance abuse (excluding caffeine and tobacco); no neurological disorder, no earlier treatment due to psychiatric disorder. They were also screened with SCID-5 to exclude the presence of a psychiatric disorder. The two groups were matched in age, sex, ethnic origin and educational status to minimize interindividual variability.

After a detailed description of the study written informed consents were obtained. Participants were aware of the study's aims and hypotheses. The investigation was performed according to institutional guidelines. Ethical perspectives were established in accordance with the latest version of the Declaration of Helsinki. The study design was approved by the Committee on Medical Ethics, University of Pécs (ethical permit number: 6539).

### Experimental task

After reading the short story “The End of Something” written by Ernest Hemingway, a structured interview was done with 14 questions. Spontaneous mental state reasoning, explicit mental state inference, comprehension of non-mental aspects of the story and the existence of previous experience in reading literary fiction were evaluated, examining whether prior knowledge of literary fiction and frequent reading literary fiction affect explicit ToM skills.

Authors applied the original Short Story Task method created by Dodell-Feder et al. (27) to observe ToM in healthy participants. The Short Story Task consists of a short story and its administration and scoring materials. This Supplementary material contains instructions and scoring materials. The original English version of the Supplementary material is downloadable from the publisher's website at: https://journals.plos.org/plosone/article?id=10.1371/journal.pone.0081279.

In this study the Hungarian version of the test was used (36). The test was translated and adapted into Hungarian. ToM skills were analyzed by means of a structured interview after reading the short story “The end of something” by Ernest Hemingway, which was chosen for its simplicity and readability (27). The story is about a breakup; however, the characters' psychological lives are not explicitly described, so the reader is forced to draw conclusions about their mental states from the indirect communication between the characters.

Prior to reading, participants were given verbal instructions and subsequently a series of open-ended questions with a particular focus on eliciting the thoughts, feelings and intentions of the characters. The task was presented verbally by one of the investigators in the form of an interview in one session for all participants individually. Each interview was recorded, and the recorded data were scored by two independent investigators. The interrater reliability was tested, and the kappa coefficient was >0.90 in the pilot study. Scoring was completed by the first author (Fekete), using the transcripts, then 25% of the transcripts were chosen at random and scored by a second independent rater (Varga).

The first question of the structured inteview is an informal discussion, the aim of which is to ask if the participant is familiar with the story. In conjunction with this question the participants were explored about their reading habits to determine if they had previous reading experience. Reading experience (e.g., actual or earlier regular reading habit) was assessed by this exploration (0 points if the subjects had no previous reading experience, 1 point if prior reading experience could be verified). After that participants were asked a single question that simply asking them to summarize the story to assess the inference of spontaneous mental state. Responses were coded for the presence (1) or absence (0) of spontaneous use of mental state inferences (27). Further instructions were then provided and participants were asked to include in their answers what they thought the characters were thinking, feeling or intending, as long as they relevant to the question. For scoring purposes each question was assigned a value of 0 (no answer or incorrect answer), 1 (partial understanding or clarifying questions were needed), or 2 (full understanding), and an overall comprehension score was calculated from the sum of points from the five comprehension questions and explicit mental state reasoning scores were calculated as the sum of points from the eight mental state reasoning questions. Thus, explicit ToM scores can range from 0—indicating little to no understanding of the story characters' mental states—to 16—indicating excellent understanding of the story characters' mental states.

### Statistical analysis

IBM SPSS Statistics version 28.0 software was used for statistical analysis. Independent samples *t*-test, correlation, Chi-square test, Fisher's Exact Test, and linear regression were used for analyses. We used the independent samples *t*-test to compare the means of the different groups (persons with schizophrenia group and the control group) for both clinical and demographic data. With the help of correlation we calculated the relationship between the prior reading and the years of education. We used the Chi-square test to examine the relationship between diagnosis and previous reading experience. Fisher's Exact Test was used to analyze the relationship between prior reading and spontaneous ToM. A series of linear regressions was carried out to estimate path models to investigate the association between prior reading, comprehension and ToM.

## Results

As we reported in our previous publication (36), there was no significant difference in age, years of education and gender ratio. The mean duration of illness was 12.46 years (SD = 2.32 years). The healthy controls achieved a better result in spontaneous and explicit ToM questions, but there was no significant difference in comprehension questions (see Table 1). Linear regression revealed that ToM was not influenced by demographic variables. Both groups showed a relatively normal distribution of explicit ToM scores without ceiling effect.

**Table 1:** Differences in task performance between the experimental groups.

**Experimental tasks**	**Control group (*****n*** = **48)**		**Schizophrenia group (*****n*** = **47)**		***p*-value**
	**Mean**	**S. D**.	**Mean**	**S. D**.	
Comprehension questions	8.42	1.76	6.55	2.34	0.050^*^ n.s.
Explicit mental state reasoning questions	9.08	3.75	4.98	3.96	< 0.001^*^ sig.
Spontaneous mental state inference question	0.27	0.54	0.09	0.28	< 0.001^*^ sig.

Independent samples t-test.

^*^Statistically significant: *p* < 0.01, Bonferroni correction, n, number; SD, standard deviation; n.s., not significant; sig, significant (36).

In the present analysis, we found a comparable number of subjects with reading experience in both groups. Twelve (25.5%) of the 47 subjects in SG and 18 (37.5%) of the 48 participants in CG were prior readers. We conducted a Chi-square test and found no relationship between diagnosis and previous reading (*p* = 0.210), so whether a person has read literary fiction in the past is independent of their diagnosis.

The correlation test showed that there is a weak positive relationship (*r* = 0.280) between the years of education completed and having read fiction in the past (*p* = 0.006). Thus, the more education was completed, the more likely it is that he/she had read fiction in the past.

A significant difference (*t* = −4.017, *p* < 0.001, M_difference_ = −1.859, CI = −2.778, −0.940) was detected in comprehension mean scores between the readers (M = 8.77, SD = 1.569) and non-readers (M = 6.91, SD = 2.296). Significant differences (*t* = 2.132, *p* = 0.019, M_difference_ = 1.607, CI = 0.089, 3.126) were found in the group of patients (readers: M = 7.75, SD = 1.765, non-readers: M = 6.14, SD = 2.39), and it was present also in healthy controls (*t* = 3.485, *p* < 0.001, M_difference_ = 1.644, CI = 0.695, 2.594; readers: M = 9.44, SD = 0.984, non-readers: M = 7.80, SD = 1.846).

Looking at the two groups in general it can be stated, that participants with prior reading experience had almost twice the average ToM scores (M = 10.07, SD = 4.102) of the non-readers (M = 5.66, SD = 3.747), and the fact that the former were statistically significantly superior to the latter was confirmed by the results of the independent samples *t*-test. We confirmed that reading literary fiction in the past is advantageous in terms of ToM. We found that at the 5% significance level, there is a significant difference between the average ToM scores of former readers and non-readers (*t* = −5.169, *p* < 0.001, M_difference_ = −4.405, CI = −6.097, −2.713).

We found that ToM scores were significantly higher among those who had prior reading experiences in the control and also in the schizophrenia group. Among patients, previous readers have more than twice the average ToM score than non-readers (see Table 2). The difference was significant (*t* = −2.944, *p* = 0.010, M_difference_ = −4.169, CI = −7.190, −1.148). Control subjects with reading experience scored also significantly higher (*t* = −3.721, *p* < 0.001, M_difference_ = −3.689, CI = −5.684, −1.693) compared to non-reader controls. As presented in Table 2, the difference between the non-reading and reading scores of participants with schizophrenia was not smaller than the previous reading and non-reading scores of healthy participants. There was a significant difference in the mean ToM scores between former readers of SG and CG subjects and non-readers of the experimental groups. The mean score of explicit ToM was significantly lower (*t* = −4.676, *p* < 0.001, M_difference_ = −3.786, CI = −5.404, −2.168) in the non-reading schizophrenia group compared to the non-reading control group. Readers of the CG group also performed significantly better (*t* = −2.320, *p* = 0.014, M_difference_ = −3.306, CI = −6.224, −0.387) in the explicit ToM compared to the readers of the SG (see Table 2).

**Table 2:** Differences in explicit ToM points in the four subgroups (readers and non-readers in both the schizophrenia and control groups).

	**Schizophrenia group**			**Control group**			*p*-value^*^
	***n***	**Mean**	**SD**	***n***	**Mean**	**SD**	
Non-readers	35	3.91	3.17	30	7.70	3.35	< 0.001 sig.
Readers	12	8.08	4.54	18	11.39	3.27	0.014 sig.
*p*-value^**^	0.01 significant			< 0.001 significant			

Independent samples t-test.

^*^p-value of the between-group analysis.

^**^p-value of the within-group analysis, sig, significant; n, number; SD, standard deviation.

Previous reading experience and spontaneous ToM ability show a medium-strength relationship (*p* < 0.001, Cramer's *V* = 0.389), those with previous reading experience are more likely to spontaneously mentalize and those without previous reading experience are more likely to fail to mentalize spontaneously (see Table 3). In the control group Chi-square test was not suitable to analyze the relationship between previous reading experience and spontaneous ToM ability, so Fisher's Exact Test was used. At all reasonable levels of significance, there was a medium significant correlation for the control group (*p* < 0.001, Cramer's *V* = 0.602), meaning that those with previous reading experience were more likely to give spontaneous ToM answers, while those without were more likely to fail to mentalize spontaneously. Fisher's Exact Test was also used in the SG to test the relationship between previous reading experience and spontaneous ToM ability. In contrast to the control group, no correlation was found in the patient group (*p* = 0.734), meaning that previous reading experience did not significantly affect spontaneous ToM ability. It should be noted; however, that the number of participants giving spontaneous ToM answers was rather small, which significantly weakens the interpretability of the results.

**Table 3:** The effect of previous reading experience on spontaneous ToM in the experimental groups.

	**Previous reading experience**	**Spontaneous ToM (** ***n*** **)**		***p*-value**
		**No**	**Yes**	
Schizophrenia group	Non-readers	32	3	0.734 n.s.
	Readers	11	1	
Control group	Non-readers	29	1	< 0.001 sig.
	Readers	8	10	

Fisher's Exact Test. n, number of subjects giving spontaneous mental state inferences; n.s., not significant; sig, significant.

**Figure 1:**
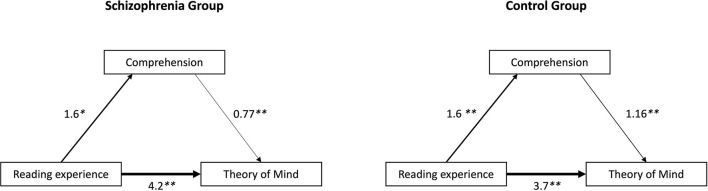
Path models of the effect of reading on comprehension and theory of mind in the experimental groups. Values on the arrows are unstandardized beta coefficients, *p < 0.05; **p < 0.001.

The path models in Figure 1 were fitted based on the results of linear regressions. All models are valid (*p* < 0.05) with explanatory power of at least 20%. For SG (see Figure 1) reading improves comprehension (*p* = 0.039), with an average increase of 1.6 points in reading comprehension if the person had former literary fiction reading experience. Previous reading also had a positive effect on ToM (*p* < 0.001), increasing its score by 4.2 points on average. Higher levels of text comprehension also improve ToM (*p* < 0.001), if text comprehension is one point higher, the model predicts an average ToM 0.8 points higher. The same can be observed for the CG (see Figure 1), but the effect sizes are slightly different; prevous reading improves comprehension (*p* < 0.001) by an average of 1.6 points and ToM (*p* < 0.001) by an average of 3.7 points on average. Furthermore, a 1.2-point improvement in the average ToM score is associated with a one-point higher score in text comprehension (*p* < 0.001). Overall, therefore, it can be concluded that prior reading experience has a partial effect on comprehension and also a direct effect on ToM, and that comprehension also has a direct impact on ToM.

## Discussion

In our current research we sought to explore the relationship between previous reading experiences and ToM skills. We hypothesized, that previous literary fiction reading experience would be associated with better explicit ToM performance. Readers scored nearly twice as high scores as non-readers, and this was true for both patients with schizophrenia, and healthy controls. Nonetheless, the explicit ToM performance of patients in the reading SG was still poorer compared to subjects in reader CG. It was also discovered that previous reading experience also had a positive impact on comprehension scores. Subjects with previous reading experience scored higher in comprehension in both SG and CG. Also interestingly, previous reading experience was weakly correlated with educational attainment as well, although in our previous analysis, educational attainment had no effect on SST performance. Furthermore, earlier readers were more likely to use spontaneous mental state terms, but this relationship was not present for SG. It should be noted; however, that the small number of subjects giving spontaneous ToM answers, especially in SG significantly reduces the generalizability of this effect. Finally, path model analysis revealed that reading experience has a bidirectional effect on ToM. It has a significantly direct positive effect on ToM, but it also improves comprehension, albeit to a lesser extent, which in turn has a direct positive impact on ToM.

Our results are in line with recent data suggesting a positive correlation between reading and ToM (8–10, 17, 27). The causative association between the two; however, is still a matter of debate. The question has already been raised in the study by Kidd and Castano (9), whether prior knowledge of fiction predicts better performance on mentalization tests, or whether prior knowledge of non-fiction (informational and documentary) literature may be an equally influential factor. It was concluded that prior knowledge of fiction is a strong predictor of ToM performance but found no such improvement for genre literature or non-fiction/documentary literature. These findings are in line with Black and Barnes' (17) research that reading fiction can lead to short-term improvements in mentalization skills, and when the data was controlled for prior reading experience, the mentalization-enhancing effect persisted. This suggests that fiction improves mentalization independent of prior experience. Black and Barnes also proposed the possibility that lifelong reading habits alone may be associated with faster causal mapping, but it is also possible that people who read literary fiction are those who are more oriented to rule-based systems, and that reading certain literary genres enhances the ability to see causal relationships, which is precisely what is needed to understand fictional stories (17). Overall, there is a spiral dynamic: those who like to read literary fiction develop and sensitize their ToM skills, and this gives them a sense of achievement, which makes them more likely to pick up literary fiction again. And those who do not or rarely read literary fiction are less likely to return to them. Consistent with Panero's conclusion that the most likely relationship between reading literary fiction and mentalization is that those with better ToM skills are more likely to read and/or that lifelong reading consistently improves mentalization ability; however, other variables (e.g., verbal ability) may also play a role (22).

Recent evidence also highlights the impact of verbal skills and vocabulary learning on ToM. A longitudinal study investigated the relationship between verbal language skills and ToM from early childhood to early adolescence (41). The development of early ToM skills was predicted by language skills, particularly receptive grammar and sentence comprehension. In addition, early ToM abilities predicted vocabulary, and the subsequently developed advanced ToM skills affected listening comprehension, which in turn improved reading comprehension. Heyes and Frith (1) suggest, that vocabulary growth is associated with the acquisition of terms that can be used to express mental states and therefore vocabulary acquisition is an important factor in the development of mental state reasoning. It is also well-documented that extensive reading can increase vocabulary acquisition (42, 43). Our findings also indicate that previous reading may enhance the ability to use mental state descriptions spontaneously.

Reading experience may also be linked to years of education, as we have shown in our analysis. Recently education, spontaneous ToM and the amount of books read were found significant predictors of explicit ToM performance (38).

Language skill impairments are well-established in schizophrenia (44, 45). Limited use of mental state terms was also reported in subjects with schizophrenia (46). Moreover, studies of children born to parents with schizophrenia have shown that an impaired subgroup of these high risk subjects exhibit marked deficits in pragmatic language skills, receptive language, explicit and spontaneous ToM as early as age of 7 (47). It seems reasonable to hypothesize that reading fiction may have a positive impact on ToM skills in subjects at risk of developing schizophrenia, or in patients already living with schizophrenia. This hypothesis is indirectly supported by the recent article mentioned, where the authors found that the number of books previously read predicted the SST performance in autistic and non-autistic individuals (38).

All of these findings suggest that reading literary fiction may be a cost-effective remediation tool that can be integrated into the rehabilitation of patients with schizophrenia (21). It has been long known that bibliotherapy and poetry therapy helps participants to address issues such as the question of identity and how we function in our social roles as well as to see how we relate to ourselves and to each other by being inspired by literary fiction (8, 48). According to Scott (49) one of the most important skills mediated by literary fiction, and perhaps to some extent by all fiction, is the ability to navigate between two mental positions, such as identifying with the characters and detaching from the story, and this negotiation is thought to be the essence of encountering narrative fiction. Therapeutic use of this kind of oscillation may support remediation of ToM skills in subjects who have difficulties in reasoning about mental states.

### Limitations

There are some limitations to our findings. Most importantly we measured that reading experience through an initial informal interview based on self-evaluated familiarity with literature and reading habits. Additionally we evaluated it dichotomously; however, a more detailed description of reading experience could have provided a more comprehensive picture of the relationship between reading and ToM. Nevertheless, our results seem to be in line with the previously cited study, where the number of books read per month was used to assess reading experiences (38). In addition to quantity, a more detailed analysis would be interesting (e.g., regular reading vs. occasional reading, what genre they read, how characteristic reading was in different developmental periods, etc.). Another limitation of our study is the lack of IQ assessment. Although the relationship between general intelligence and reading habits is a poorly studied area, we cannot rule out even a possible bidirectional relationship between IQ and reading, which in turn may influence ToM skills (50, 51). In any case, as our paper is a re-analysis of a previous study, it still has the same limitations as the original publication, namely the lack of full PANSS, the absence of other measures to assess the concurrent validity of SST. We also need to point out, that patients were on maintenance medication, which could have affected patients' performance (36). Studies of the effects of psychotropic medications on social cognition have not yielded clear results (52). However, current data suggest that antipsychotics do not appear to have a significant impact on ToM performance (53) and in a recent study of patients with bipolar and schizophrenia spectrum disorders, antipsychotics, antidepressants and benzodiazepines showed no significant effect on ToM (54).

Future studies should address the limitations of our findings. Firstly, a more rigorous description of the reading experience would provide more reliable data on the effect of reading on ToM performance, but a direct investigation of the effect of reading literary fiction with baseline and post intervention assessment would be even more informative. It would also be valuable to better understand how general intelligence and specific neurocognitive skills influence and interfere with reading abilities. Clarification of these questions is inevitable for a possible future therapeutic application of reading literary fiction in the rehabilitation of patients with schizophrenia.

## Conclusions

In conclusion it could be stated that previous reading experience is associated with better text comprehension and ToM performance in SST. Previous reading experience can have a direct effect on ToM and can also have an indirect effect through improving text comprehension. These results can be demonstrated not only in healthy subjects, but also in individuals living with schizophrenia. Overall we conclude that there is a significant difference between readers and non-readers and we therefore interpret, that this may be improved through empathy, attunement, identification skills and intersubjective relatedness, following model behavior and developing new schemas. This suggests that regular reading has long-term beneficial consequences, as it not only improves text comprehension skills but may also enhance mentalization skills.

All these findings and interpretations are in line with the results of several recent studies on this topic (55–57) which show that reading (fiction) for longer periods of time, even throughout life, is associated with higher mentalizing abilities. This may be particularly true for cognitive ToM and although reading a particular work of literary fiction may not have always an immediately measurable positive effect on mindreading abilities, lifelong reading may be associated with more advanced mentalizing abilities. This, in turn, emphasizes the therapeutic potential of integrating the reading of literary fiction into the remediation of cognitive skills in patients living with schizophrenia.

## Data availability statement

The raw data supporting the conclusions of this article will be made available by the authors, without undue reservation.

## Ethics statement

The studies involving human participants were reviewed and approved by Regional Research Ethics Committee, Pécs. The patients/participants provided their written informed consent to participate in this study.

## Author contributions

JF: study design, data collection, interview scoring, and manuscript writing. ZP: statistical analysis and manuscript writing. EV: interview scoring and data analysis. DH, MH, NA, and BP: data collection and data analysis. TT: psychopathology assessment and manuscript revision. RH: study design, psychopathology assessment, manuscript writing, and revision. All authors contributed to the article and approved the submitted version.
